# From calcium pump to metabolic hub: emerging genetic phenotypes and metabolic networks of SERCA2 in skeletal muscle

**DOI:** 10.3389/fphys.2026.1775444

**Published:** 2026-03-19

**Authors:** Shunyi Lei, Yanlong Qu, Jin Yan, Fei Nan, Siyao Liu, Wenhao Pan, Chaoyue Yu

**Affiliations:** 1 Third Department of Orthopedics, The First Affiliated Hospital of Harbin Medical University, Harbin, China; 2 Department of Trauma, Hand, Foot and Ankle Surgery, Baotou City Fourth Hospital, Baotou, China

**Keywords:** ATP2A2, ER stress, metabolic hub, non-shivering thermogenesis, SERCA2, skeletal muscle

## Abstract

For decades, the sarco/endoplasmic reticulum Ca^2+^ ATPase 2 (SERCA2) in skeletal muscle was primarily recognized for its role in orchestrating slow-twitch muscle fiber relaxation—an essential process dependent on its ability to actively sequester cytoplasmic Ca^2+^ into the sarcoplasmic reticulum (SR) lumen, thereby sustaining intracellular Ca^2+^ homeostasis critical for muscle contraction-relaxation cycles. However, recent genetic and molecular biology studies have expanded the function of SERCA2 to a core hub integrating Ca^2+^ signaling, metabolic homeostasis, and endoplasmic reticulum (ER) stress. This novel function is underpinned by a sophisticated multi-layered regulatory network spanning from transcription to post-translational, which ensures that SERCA2 expression and activity dynamically adapt to the dual demands of Ca^2+^ homeostasis maintenance and metabolic signaling demands. Dysregulation of this network or mutations in the ATP2A2 gene have been linked to hereditary myopathies, while SERCA2 dysfunction is also a key driver of muscle atrophy and insulin resistance in pathological conditions such as chronic inflammation and obesity. As a metabolic hub, the core mechanism of SERCA2 lies in its role as a critical node connecting local Ca^2+^ signaling to systemic metabolism through regulating ER Ca^2+^ homeostasis and SERCA2-SLN uncoupling (mediating non-shivering thermogenesis). Therapeutic strategies targeting SERCA2, including small-molecule activators such as CDN1163, AAV9-SERCA2a gene therapy, mimetic peptides, and exercise interventions, have demonstrated potential in treating various systemic diseases by restoring the “calcium pump-metabolism” dual functions of SERCA2. However, the hierarchical regulatory logic linking SERCA2’s calcium-handling and metabolic functions remains fragmented, and subtype-specific therapeutic strategies are undefined. This review synthesizes recent breakthroughs to propose a unified “calcium-metabolism coupling” framework and identifies translational gaps for precision targeting.

## 1 From traditional calcium pump to metabolic hub

The sarco/endoplasmic reticulum Ca^2+^ ATPase (SERCA) family, as core members of the P-type ATPase superfamily, is evolutionarily conserved in eukaryotes. Its core function is to actively transport cytoplasmic Ca^2+^ into the sarcoplasmic/endoplasmic reticulum (SR/ER) lumen against the electrochemical gradient, thereby maintaining intracellular Ca^2+^ homeostasis (Valentim et al., 2022). This Ca^2+^ sequestration process is not only a critical link in the muscle contraction-relaxation cycle but also regulates multiple signaling pathways with Ca^2+^ as the second messenger, including cell proliferation, differentiation, and energy metabolism. Phylogenetic analyses reveal three principal SERCA isoforms—SERCA1, SERCA2, and SERCA3—exhibiting fundamentally divergent expression patterns across tissue types (Zhihao et al., 2020): SERCA1 is predominantly expressed in fast-twitch muscle fibers, SERCA3 is widely present in non-muscle tissues, while SERCA2 (encompassing two key subtypes, SERCA2a and SERCA2b) exhibits the most diverse functions—SERCA2a is mainly distributed in the heart and slow-twitch muscle fibers, and SERCA2b is ubiquitously expressed, serving as a core subtype involved in physiological adaptation and pathological progression (Pant et al., 2016; Salag et al., 2024). The functional characteristics of SERCA2 subtypes are also evolutionarily conserved with species-specificity in non-mammals. For example, in zebrafish (*Danio rerio*), knockout of the atp2a2a gene leads to abnormal skeletal muscle morphology and impaired locomotor capacity, and this functional defect cannot be rescued by mutant SERCA2a, highlighting the critical role of this subtype in skeletal muscle development and function (Malaichamy et al., 2025). Additionally, in Japanese medaka (*Oryzias latipes*) subjected to cold adaptation (exposure to 4 °C), the transcriptional level of SERCA2 in skeletal muscle is significantly upregulated, and it synergizes with SERCA1 to mediate non-shivering thermogenesis, revealing its role in adaptive energy metabolism (Robinson et al., 2024). These cross-species studies not only confirm the central position of SERCA2 in skeletal muscle physiological functions but also imply that its metabolic regulatory role is evolutionarily conserved, laying the foundation for subsequent functional expansion research.

Despite the well-established role of SERCA2 as a core calcium pump, emerging genetic, molecular biology, and physiological studies are gradually uncovering its profound significance beyond the traditional role of a Ca^2+^ transporter. In particular, SERCA2 is not only a key enzyme maintaining cellular Ca^2+^ homeostasis but also redefined as a core hub integrating Ca^2+^ signaling, metabolic remodeling, and ER stress response (Periasamy et al., 2017; Xu and Van Remmen, 2021). This paradigm shift stems from the in-depth analysis of its sophisticated multi-layered regulatory network (Ren et al., 2024; Umar et al., 2025; Deng et al., 2025), as well as the dual pathological consequences of its dysfunction or ATP2A2 gene mutations—hereditary myopathies and systemic metabolic disorders (Malaichamy et al., 2025; Llansó et al., 2025). This review aims to integrate these breakthrough advances, establish a unified “calcium-metabolism coupling” theoretical framework, and systematically clarify the novel role of SERCA2 in skeletal muscle. Following the logical chain of “regulatory basis → functional abnormalities → metabolic expansion → therapeutic repair,” we will delve into the fine regulatory network of SERCA2, the pathological characteristics induced by its dysfunction, its critical role in metabolic remodeling (especially thermogenic mechanisms), and potential therapeutic strategies targeting SERCA2, with the goal of providing an integrated and forward-looking perspective for research in this field.

## 2 Multi-layered regulatory network of SERCA2 in skeletal muscle

**FIGURE 1:**
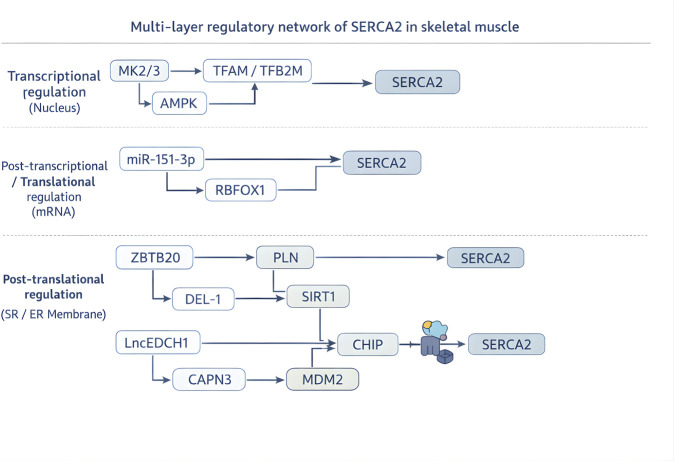
Multi-layer regulatory network of SERCA2 in skeletal muscle.

To simultaneously meet the demands of “Ca^2+^ homeostasis maintenance” and “metabolic signal response” in skeletal muscle, the molecular basis of SERCA2 lies in a multi-layered regulatory network from transcription to protein-protein interaction—this network not only ensures the dynamic balance of SERCA2 expression and activity but also constructs cross-talk pathways between “Ca^2+^ signaling” and “metabolic signaling”, providing a mechanistic basis for functional evolution (Table 1; Figure 1).

**TABLE 1:** Summary of the multi-layered regulatory network of SERCA2 subtypes in skeletal muscle.

Subtype	Core pathway	Regulatory mechanism	Related diseases	References
SERCA2a	p38 MAPK-MK2/3-Atp2a2	MK2/3 binds to the AP-1 site in the Atp2a2 promoter to inhibit transcription, while AMPK inhibits MK2/3 to relieve this suppression	Involved in abnormal myofiber type transition	Scharf et al. (2013)
	RBFOX1-Serca2a mRNA	RBFOX1 binds to the UGCAUG element in the 3′-UTR of Serca2a mRNA, enhancing the translation of SERCA2a	Cardiac hypertrophy, heart failure	Umar et al. (2025)
	ZBTB20-PLN	ZBTB20 indirectly inhibits PLN transcription; the reduction of PLN relieves the inhibition on SERCA2a	No direct diseases in skeletal muscle (causes abnormal myocardial contractile function)	Ren et al. (2024)
	SLN Uncoupling - PGC1α	Melatonin activates the CaMKII-AMPK-PGC1α pathway, upregulating SERCA2a and SLN, and promoting their uncoupling	Obesity, metabolic syndrome	Salag et al. (2024)
	Ubiquitin-Proteasome (CAPN3-MDM2)	CAPN3 deficiency increases the binding of SERCA2a to MDM2, accelerating the K48-linked ubiquitination and degradation of SERCA2a	Limb-Girdle Muscular Dystrophy Type 1 (LGMDR1)	Lasa-Elgarresta et al. (2022)
	ATP2A2 Mutation-Related Regulation	ATP2A2 mutations (c.1583G>A/c.1117G>A) lead to decreased SERCA2a activity or abnormal intracellular localization	Recurrent rhabdomyolysis, vacuolar myopathy	Malaichamy et al. (2025)
	AAV9-SERCA2a Therapy-Related Regulation	AAV9 vector delivers the SERCA2a gene systemically, enabling continuous expression for 18 months to restore calcium homeostasis	Duchenne Muscular Dystrophy (DMD), DMD-related cardiomyopathy	Wasala et al. (2020)
	Pin1-SERCA2a	Prolyl isomerase Pin1 directly binds to SERCA2a in rat skeletal muscle to enhance its calcium transport activity	Insulin resistance, decreased exercise capacity	Nakatsu et al. (2024)
	O-GlcNAcylation-PLN	O-GlcNAcylation modification of SERCA2a decreases in hibernating Spermophilus dauricus, reducing its binding to PLN	Skeletal muscle atrophy (protective effect during hibernation)	Dang et al. (2025)
	CDN1163-SERCA2a	Small molecule CDN1163 activates SERCA2a to maintain calcium homeostasis in unloaded rat skeletal muscle	Disuse-induced skeletal muscle atrophy	Zaripova et al. (2025)
	SERCA2-SLN Cold Adaptation	Cold exposure upregulates SERCA2 transcription in *Oryzias latipes* skeletal muscle, synergizing with SLN for non-shivering thermogenesis	Impaired cold-induced thermogenesis	Robinson et al. (2024)
	Asprosin-PKCδ	Asprosin activates PKCδ, downregulating SERCA2b expression and causing endoplasmic reticulum (ER) calcium depletion	Obesity-related insulin resistance	Jung et al. (2019)
SERCA2b	Jaceosidin-SERCA2b	Jaceosidin upregulates SERCA2b, restoring ER calcium homeostasis to alleviate ER stress	Type 2 diabetes mellitus	Ouyang et al. (2017)
	DEL-1-SIRT1	DEL-1 enhances SIRT1 activity, reducing the acetylation of SERCA2b to improve protein stability	High-fat diet-induced obesity	Sun et al. (2020)
	LncEDCH1-SERCA2b	LncEDCH1 interacts with SERCA2b in mouse C2C12 myotubes to improve mitochondrial function and reduce ER stress	Denervation-induced skeletal muscle atrophy	Cai et al. (2022)
	ER Stress-Mitochondrial Dysfunction	SERCA2b deficiency triggers ER stress, which further impairs mitochondrial respiratory chain complex IV activity	Skeletal muscle metabolic disorder, atrophy	Cai et al. (2022)
	JAK2-STAT3-SERCA2a/SLN	Activated JAK2-STAT3 pathway downregulates SERCA2a and upregulates SLN in human asthmatic skeletal muscle	Skeletal muscle wasting in asthma	Qaisar et al. (2021)

### 2.1 Transcriptional regulation

At the transcriptional level, MK2/3, downstream effectors of the p38 MAPK pathway, are core repressors of the Atp2a2 gene encoding SERCA2a (Shiryaev and Moens, 2010). ChIP assays confirm that MK2/3 can directly bind to the AP-1 binding site in the Atp2a2 promoter to inhibit its transcription (Scharf et al., 2013); in MK2/3 knockout mice, SERCA2a expression is upregulated by 2.1-fold, accompanied by the conversion of fast-twitch muscle fibers to slow-twitch muscle fibers, and the synchronous upregulation of the slow-twitch muscle marker gene MyHC I by 1.8-fold. The physiological significance of this regulation is that slow-twitch muscle fibers are the main site where SERCA2 exerts metabolic functions (such as non-shivering thermogenesis) (Deng et al., 2025). By “braking” Atp2a2 transcription, MK2/3 achieves the matching of “muscle fiber type - SERCA2 function”—when the body needs to enhance thermogenesis, MK2/3 activity is inhibited by AMPK, and the upregulation of SERCA2a can rapidly improve the metabolic adaptability of slow-twitch muscle fibers.

The transcriptional regulation mediated by MK2/3 suggests the adaptation mechanism between SERCA2 expression and muscle fiber metabolic characteristics, while the involvement of mitochondrial transcription factors adds a new dimension of interorganellar coordination to its regulatory network. In addition to the p38 MAPK-MK2/3 pathway, the nuclear regulation of SERCA2 by mitochondrial transcription factors provides a novel perspective on interorganellar coordination. Watanabe et al. (2011). found in cardiomyocytes that TFAM and TFB2M can bind to the SERCA2 gene promoter region (the GGGGGCGGG sequence at positions −2122 to −2114), increasing transcriptional activity by 1.76–2.02-fold. Whether a similar TFAM/TFB2M-dependent regulatory mechanism operates in skeletal muscle remains to be determined.

### 2.2 Post-transcriptional regulation

At the post-transcriptional level, microRNAs (miRNAs) achieve dynamic fine-tuning of SERCA2 through targeted regulation. Notably, miR-151-3p governs SERCA2a expression through direct targeting of a conserved seed region (nucleotides 1245-1251) within the Atp2a2 3′-UTR, suppressing translational output by approximately 40% in both C2C12-derived myotubes and primary skeletal muscle fibers. This post-transcriptional repression coordinately attenuates the slow-fiber transcriptional program, exemplified by reduced MyHC I and PGC-1α levels (Wei et al., 2015).

Functional experiments confirmed that this microRNA regulatory mechanism enables the functional switching of SERCA2 under stress conditions. After acute exercise stress (such as mouse exhaustive treadmill exercise), the expression of miR-151-3p in skeletal muscle is upregulated by 2.3-fold, the protein level of SERCA2a is decreased by 35%, and the contraction speed of fast-twitch muscle fibers is increased by 20%, indicating that this regulation can prioritize the rapid contraction function of fast-twitch muscles; during cold adaptation (7-day exposure to 4 °C), the expression of miR-151-3p is downregulated by 50%, and SERCA2a expression is restored, laying the molecular foundation for non-shivering thermogenesis (Pant et al., 2016; Wei et al., 2015).

Furthermore, the RNA-binding protein RBFOX1 has been reported to bind SERCA2 (Atp2a2) mRNA and enhance translation efficiency, thereby increasing SERCA2 protein synthesis (Umar et al., 2025). RBFOX1 expression is coupled to cellular energy sensing pathways, such as AMPK signaling, suggesting a potential link between energy status and SERCA2 translational output (Sjåland et al., 2011; Maurya and Periasamy, 2015).

Notably, some long non-coding RNAs influence SERCA2 abundance mainly by modulating protein stability (e.g., via ubiquitination) and are therefore discussed in Section 2.3.

### 2.3 Post-translational modifications and protein-protein interactions

In addition to the classical regulation by phospholamban (PLN), newly discovered regulatory proteins in recent years have further expanded the functional network of SERCA2. In the heart, ZBTB20 can indirectly enhance SERCA2a activity by downregulating PLN expression (Ren et al., 2024), while in skeletal muscle, this regulatory axis is associated with insulin signaling. When stimulated by insulin, the upregulation of ZBTB20 can accelerate Ca^2+^ reuptake by activating SERCA2a, thereby promoting muscle glycogen synthesis and achieving the coordination of “Ca^2+^ signaling - metabolic signaling”. Meanwhile, Developmental endothelial locus-1 (DEL-1) can enhance SIRT1 expression, reduce the acetylation modification of SERCA2 protein, and improve its stability (Sun et al., 2020). In the skeletal muscle of high-fat diet-induced obese mice, decreased DEL-1 expression leads to SIRT1 inactivation and SERCA2 degradation, inducing both Ca^2+^ homeostasis imbalance and exacerbated insulin resistance through ER stress (Sun et al., 2020; Jung et al., 2019). This mechanism suggests that post-translational modifications of SERCA2 not only regulate calcium pump activity but also are linked to metabolic homeostasis, supporting its role as a “metabolic hub”.

In 2022, Cai et al. (2022) reported that LncEDCH1 expression positively correlates with SERCA2 protein abundance. Mechanistically, LncEDCH1 directly binds the N domain of SERCA2 via its 3′-terminal 218–326 nt region, attenuates the interaction between SERCA2 and the E3 ubiquitin ligase CHIP, reduces K48-linked ubiquitination, and thereby prolongs SERCA2 protein half-life. In a denervation-induced atrophy model, LncEDCH1 overexpression partially restored SERCA2 activity, activated the AMPK–PGC-1α axis, improved mitochondrial complex IV activity, and mitigated myofiber atrophy.

Moreover, in hibernating Daurian ground squirrels (Spermophilus dauricus), Dang K et al. reported that reduced O-glycosylation of SERCA2a during hibernation decreases its association with PLN, thereby helping preserve calcium pump activity and potentially mitigating disuse-associated muscle atrophy (Dang et al., 2025). Collectively, these post-translational mechanisms support the notion that SERCA2 regulation is deeply integrated with metabolic signaling, consistent with its proposed “metabolic hub” role.

In 2022, Lasa-Elgarresta et al. (Qaisar et al., 2021) described a ubiquitin–proteasome degradation mechanism for SERCA2a. In human LGMDR1 (CAPN3-mutated myopathy), CAPN3 deficiency increases the binding of SERCA2a to the E3 ubiquitin ligase MDM2, promoting K48-linked ubiquitination modification of SERCA2a and accelerating its degradation (a 55% decrease in SERCA2a protein level). The proteasome inhibitor bortezomib can significantly reverse this process: in human CAPN3-deficient myotubes, bortezomib treatment restores SERCA2 protein level by 70%, reduces cytoplasmic Ca^2+^ concentration from 180 nmol/L to 120 nmol/L, and improves myotube contraction function by 40%.

Isoform-specific structural features of SERCA2 can tune pump kinetics in response to ER Ca^2+^ load and thereby influence metabolic signaling. For example, as a widely expressed SERCA2 subtype in skeletal muscle, Inoue (Inoue et al., 2019) found through crystal structure analysis that compared with the muscle fiber-specific SERCA2a, SERCA2b has an additional 49-amino-acid extension at its C-terminus, forming the TM11 transmembrane helix and luminal tail. TM11 provides the structural basis for endogenous active autoinhibition, a mechanism that plays an important fine-tuning role in skeletal muscle metabolic remodeling. TM11 restricts the conformational movement of the transmembrane helix bundle domain through weak interactions with TM10 and the L8/9 loop, ultimately inhibiting the catalytic cycle; this “brake” mechanism allows SERCA2b in skeletal muscle to dynamically regulate activity according to ER Ca^2+^ load: when ER Ca^2+^ concentration exceeds 600 μM, the weak interaction between TM11 and the main domain reversibly dissociates, switching SERCA2b to a “high-turnover” mode. Mutation of F1018G/V1029G can mimic this dissociated state, increasing the ATP consumption rate of SERCA2b in skeletal muscle by 70%, thereby activating the AMPK-PGC1α pathway and promoting mitochondrial biogenesis. This structural regulation explains why the activity of SERCA2b in skeletal muscle can be increased by 40% through dynamic rearrangement of TM11 after endurance training without changes in expression level, to meet the metabolic demands of continuous Ca^2+^ cycling.

## 3 SERCA2 dysfunction: pathophysiological consequences and metabolic remodeling

For a long time, ATP2A2 mutations were thought to be only associated with Darier disease (keratosis follicularis), but studies from 2024 to 2025 have confirmed that specific ATP2A2 mutations can directly cause primary skeletal muscle diseases—these findings not only fill the gap in the genetic diagnosis of skeletal muscle diseases but also verify the “function dependence” of SERCA2 at the pathological level: calcium pump dysfunction is a direct cause of myopathies, while impaired metabolic function exacerbates the pathological process.

### 3.1 SERCA2 mutations and inherited myopathies

**FIGURE 2:**
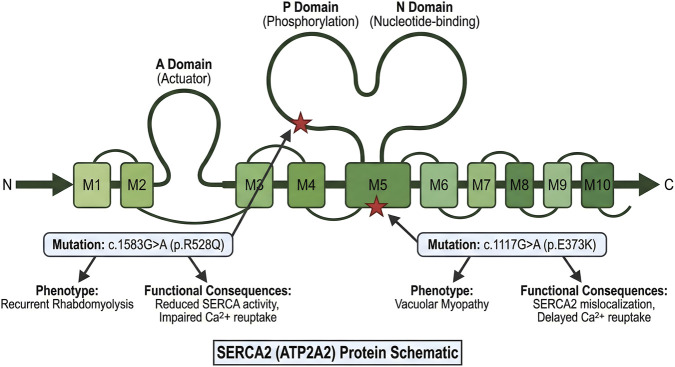
Map of ATP2A2 (SERCA2) mutations associated with inherited skeletal muscle phenotypes.

In 2025, Malaichamy et al. (2025) found that a heterozygous missense variant of ATP2A2 (c.1583G>A, p. R528Q) can cause autosomal dominant recurrent rhabdomyolysis. Experiments showed that functional characterization demonstrated that this variant attenuates Ca^2+^ transport capacity of human SERCA2a by 45%, impeding cytosolic Ca^2+^ sequestration in slow-twitch fibers under physiological stress, thereby triggering calpain-mediated proteolysis and apoptotic signaling cascades (Figure 2).

Clinical data indicated that all patients carrying this mutation had unexplained episodes of rhabdomyolysis, with significantly elevated serum creatine kinase levels (mean 5600 U/L). This finding suggests that ATP2A2 should be included in the gene screening panel for unexplained rhabdomyolysis. Llansó et al. (2025) recently identified a homozygous missense ATP2A2 variant (c.1117G>A, p. Glu373Lys) as the causal factor for a previously unreported adult-onset vacuolar myopathy. Functional assays revealed that this mutation impairs SERCA2a Ca^2+^ reuptake activity by 72%, leading to prolonged cytoplasmic Ca^2+^ accumulation in type I muscle fibers. This Ca^2+^ dyshomeostasis triggers persistent ER stress and subsequent vacuolar formation—consistent with our observation that SERCA2 dysfunction directly links Ca^2+^ imbalance to myopathic phenotypes. Clinically, this finding expands the genetic spectrum of ATP2A2-related skeletal muscle diseases, supporting the inclusion of ATP2A2 in diagnostic panels for adult-onset vacuolar myopathies.

Meanwhile, SERCA2 dysfunction is a key driver of muscle atrophy in chronic inflammatory diseases. Gonnot F (Llansó et al., 2025) found that the phosphorylation of SERCA2a at Ser663 is significantly increased in human ischemic skeletal muscle; inhibiting phosphorylation at this site can enhance the Ca^2+^ transport activity of SERCA2a by 40%, reduce cytoplasmic Ca^2+^ overload, and slow disease progression. In addition, a study by Qaisar et al. (2021) on male asthma patients aged 58-72 years showed that compared with healthy individuals, the expression of SERCA2a protein in the quadriceps of asthma patients was decreased by 42%, SERCA activity was reduced by 38%, accompanied by a 2.1-fold upregulation of SLN (a SERCA inhibitor). Mechanistically, the plasma level of periostin (an inflammatory factor) in asthma patients was increased by 3.5-fold, which inhibits SERCA2 transcription (a 40% decrease in Atp2a2 mRNA) by activating the JAK2-STAT3 pathway, while enhancing oxidative stress (a 2.8-fold increase in MDA). This suggests that “anti-inflammation + SERCA activation” may improve muscle function in asthma patients.

### 3.2 Calcium dysregulation, ER stress, and metabolic disorders

**FIGURE 3:**
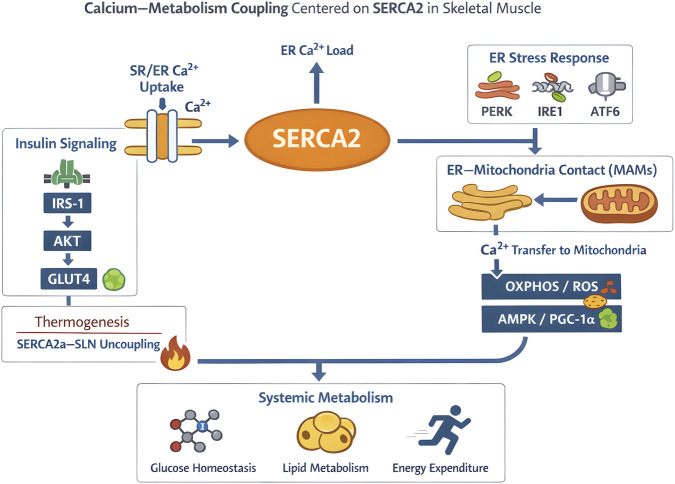
Calcium–metabolism coupling model centered on SERCA2 in skeletal muscle.

The metabolic regulatory function of SERCA2 is essentially an adaptive extension of its calcium pump function in the “energy metabolism scenario”—through two core mechanisms, “ER stress regulation” and “calcium pump uncoupling”, SERCA2 is involved in the maintenance of insulin sensitivity and non-shivering thermogenesis, respectively, serving as a critical node connecting local Ca^2+^ signaling in skeletal muscle to systemic metabolism (Figure 3).

Beyond ER-local UPR signaling, altered ER Ca^2+^ handling also reshapes ER–mitochondria communication at mitochondria-associated membranes (MAMs). At these contact sites, SERCA2 activity influences ER Ca^2+^ content and thereby the driving force for Ca^2+^ transfer from the ER to mitochondria, which can tune mitochondrial oxidative phosphorylation and redox balance (Csordás and Hajnóczky, 2009; Lynes et al., 2013). Within a physiological range, mitochondrial Ca^2+^ uptake helps match ATP supply to demand by stimulating Ca^2+^-sensitive TCA dehydrogenases and supporting oxidative metabolism (Lai and Qiu, 2020). Consistent with this concept, impaired ER–mitochondria coupling and disrupted MAM integrity have been reported in insulin-resistant skeletal muscle, supporting the functional relevance of this inter-organelle axis for metabolic homeostasis.

SERCA2b dysfunction-induced ER Ca^2+^ depletion initiates ER stress (Jung et al., 2019), which exacerbates skeletal muscle insulin resistance via three interconnected molecular pathways. First, PERK (protein kinase R-like endoplasmic reticulum kinase) activation promotes FOXO at serine residues (Copps et al., 2010), thereby contributing to insulin resistance. Second, IRE1α (inositol-requiring enzyme 1α) activation triggers its endoribonuclease activity, leading to JNK (c-Jun N-terminal kinase) phosphorylation and subsequent suppression of glucose transporter 4 (GLUT4) membrane translocation (Hage Hassan et al., 2012). Third, ATF6 (activating transcription factor 6) cleavage and nuclear translocation upregulate genes involved in lipid synthesis (e.g., SREBP1c), promoting intramyocellular lipid accumulation and impairing insulin signaling cascades (Han and Kaufman, 2016). Ouyang et al. (2017) confirmed that the natural flavonoid Jaceosidin can upregulate SERCA2b expression, restore ER Ca^2+^ homeostasis, and improve insulin sensitivity in diabetic mice (a 35% increase in ISI). The discovery of this “ER Ca^2+^ homeostasis - SERCA2b - insulin resistance” axis highlights an important role of SERCA2 in metabolic diseases, while its mediated non-shivering thermogenesis mechanism reveals another critical dimension of energy metabolism regulation.

### 3.3 Role in non-shivering thermogenesis and energy expenditure

Research on the regulation of non-shivering thermogenesis by SERCA2 in skeletal muscle is also advancing. Salagre (Salag et al., 2024) found in Zucker Diabetic Fatty (ZDF) rats that chronic melatonin treatment (10 mg/kg/d for 12 weeks) can upregulate SERCA2a expression by 50% and activity by 45% in skeletal muscle, while increasing SLN protein level by 2.3-fold, promoting non-shivering thermogenesis mediated by SERCA2a-SLN uncoupling. This is because melatonin activates CaMKII through MT1/MT2 receptors, which in turn activates AMPK, ultimately upregulating PGC1α expression by 1.6-fold. PGC-1α has also been reported to promote SERCA2a expression, which may link melatonin-induced AMPK–PGC-1α signaling to the SERCA2a upregulation described here (see also Section 4.3). Functional validation showed that melatonin treatment increased the skeletal muscle skin temperature of ZDF rats by 1.2 °C and reduced white adipose tissue weight by 32%, providing a new target for non-pharmacological intervention in obesity. Meanwhile, Zheng et al. (2025) found in a 7-day sleep deprivation model of C57BL/6J mice that sleep deprivation activates the AMPK signaling pathway in skeletal muscle, upregulates the expression of SERCA2 and UCP3, and jointly enhances adaptive thermogenesis through SERCA2 uncoupling and UCP3 mitochondrial uncoupling. Sleep-deprived mice had a 18% decrease in body fat rate and a 22% increase in resting metabolic rate (RMR), and SERCA2a knockdown completely blocked this effect, suggesting that SERCA2a is a key molecule in metabolic adaptation to sleep disorders. In addition, plant extracts can also participate in regulating SERCA2-mediated non-shivering thermogenesis. Abdillah AM (Abdillah et al., 2025) found that treatment of mouse C2C12 myocytes with capsaicin (10 μM) can upregulate SERCA2a expression by 50% through activating TRPV1 and β2-AR, promoting SERCA2a-SLN uncoupling, and increasing cellular thermogenesis by 32%. These examples illustrate the important role of SERCA2 *in vivo* metabolism.

## 4 Emerging therapeutic strategies targeting SERCA2

**FIGURE 4:**
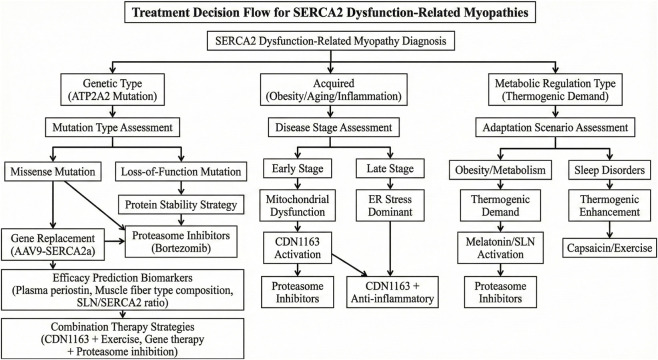
Treatment decision flow for SERCA2 dysfunction-related myopathies.

Based on the core position of SERCA2 in skeletal muscle pathology, therapeutic strategies targeting SERCA2 have developed rapidly in recent years—from small-molecule drugs that directly activate calcium pump activity, to gene therapy that repairs gene expression, and lifestyle interventions that improve the regulatory network, these strategies all achieve therapeutic effects on multiple systemic diseases by restoring the “calcium pump-metabolism” dual functions of SERCA2. Moreover, therapeutic strategies targeting SERCA2 have shown broad-spectrum potential in multi-species models (Figure 4).

### 4.1 Small molecule activators and protectors

CDN1163 is an allosteric SERCA activator with broad-spectrum therapeutic potential, which enhances catalytic efficiency by binding to the N-P domain interface of SERCA (Nogami et al., 2021). In aging models, Qaisar et al. (2020) found that CDN1163 can restore SERCA2a activity by 45% in aged mice, reversing muscle atrophy (a 12% increase in soleus muscle mass); in disuse atrophy models, Zaripova KA (Zaripova et al., 2025) found that treatment of unloaded rat skeletal muscle with 1 μM CDN1163 for 3 days can maintain SERCA2a activity and prevent increases in cytosolic Ca^2+^ levels and ATP levels; in diabetes models, Kimura et al. (2022) confirmed that CDN1163 improves insulin resistance (a 42% increase in ISI) and restores vascular endothelial function. Additionally, new studies show that in MetSyn rats, CDN1163 treatment restores SERCA2a activity by 60% in mesenteric lymphatic vessels and increases lymph flow by 45% (Lee et al., 2020); in SMA model mice, CDN1163 partially restores myocardial SERCA2a activity by 40% and extends survival by 15% (Khayrullina et al., 2020). These cross-disease and cross-organ studies together support the therapeutic potential of CDN1163 as a broad-spectrum SERCA activator, while proteasome and HDAC inhibitors provide complementary strategies at the levels of protein stability and gene expression.

The protective effect of proteasome inhibitors on SERCA2a has been verified in both human myotubes and mouse models: in addition to bortezomib, treatment of human CAPN3-deficient myotubes with carfilzomib (100 nM) can restore SERCA2a protein level by 68%, reducing cytoplasmic Ca^2+^ concentration from 180 nmol/L to 130 nmol/L (Lasa-Elgarresta et al., 2022). Furthermore, Neault N (Neault et al., 2023) found that in Myotonic Dystrophy Type 1 (DM1) models, the HDAC inhibitor vorinostat can improve skeletal muscle dysfunction in DM1 mice by upregulating the expression of SERCA1 and SERCA2a.

### 4.2 Gene therapy and RNA-based approaches

While small-molecule drugs mainly exert effects by directly regulating SERCA2 activity, gene and RNA therapies focus on repairing its expression and stability to achieve more precise targeted intervention. Significant progress has been made in the application of SERCA2a gene therapy in skeletal muscle diseases. Wasala et al. (2020) confirmed in mdx mice (a DMD model) that a single systemic injection of AAV9-SERCA2a can maintain SE.

RCA2a expression for 18 months, improving cardiac function (left ventricular ejection fraction increased from 38% to 55%) and skeletal muscle strength (grip strength increased by 38%).

Based on the protective effect of LncEDCH1 on SERCA2, Cai et al. (2022) designed an LncEDCH1 mimetic peptide (covering the 218-326 nt functional region) that binds SERCA2a and competitively blocks its interaction with CHIP and reducing ubiquitination degradation. In denervated muscle atrophy mice, intramuscular injection of the mimetic peptide restored SERCA2 protein level by 65%, increased soleus muscle mass by 12%, and improved grip strength by 18%, providing a “non-protein targeting” new approach for the treatment of muscle atrophy.

### 4.3 Lifestyle interventions and multi-dimensional approaches

Gene and drug interventions provide precise means for disease treatment, while lifestyle interventions offer practical options for prevention and adjuvant therapy, among which the effect of exercise intervention is closely related to intensity. Regarding exercise intervention, Ferreira (Ferreira et al., 2010) found that maintaining moderate-intensity aerobic exercise in mice can activate the PGC1α-MEF2 pathway, upregulating SERCA2a expression by 40%; however, the latest study on elite human rowers showed that although high-intensity training increases SR Ca^2+^ transport rate, it decreases SERCA2 expression by 18% and upregulates SERCA1 by 25% (Mazza et al., 2025), suggesting that exercise intensity needs to be personalized according to muscle fiber type.

## 5 Conclusion and future perspectives

### 5.1 Conclusion

In summary, the role of SERCA2 in skeletal muscle has expanded from a single Ca^2+^ transporter to a key metabolic hub coordinating Ca^2+^ homeostasis, ER stress, and energy metabolism. On one hand, it is finely regulated by multiple pathways at the transcriptional, translational, and post-translational levels, including RBFOX1, LncEDCH1, ZBTB20, the DEL-1–SIRT1 axis, and ubiquitination, enabling it to dynamically adapt to Ca^2+^ reuptake and metabolic demands under different physiological and pathological conditions. On the other hand, ATP2A2 gene mutations and acquired injuries such as inflammation and obesity together support that SERCA2 dysfunction not only directly induces intracellular Ca^2+^ imbalance and myopathic phenotypes in skeletal muscle but also exacerbates insulin resistance, disrupts systemic metabolic homeostasis through ER stress, and is closely associated with “calcium pump uncoupling” and non-shivering thermogenesis. Existing clinical and basic research suggests that targeted restoration or moderate enhancement of SERCA2 function (such as proteasome inhibition, activation of the AMPK–RBFOX1 and LncEDCH1 pathways, and improvement of SERCA2b expression or stability) may reverse insulin resistance and metabolic syndrome-related phenotypes while improving muscle atrophy and motor function.

### 5.2 Future research directions and translational perspectives

Although this review systematically clarifies the critical role of SERCA2 as a hub for Ca^2+^ homeostasis and metabolism in skeletal muscle, there are still many open questions in this field, leaving substantial room for future work. In particular, given the homology of SERCA2 in different tissues, drawing on and verifying the physiological and pathological significance of SERCA2 regulatory mechanisms identified in other tissues in skeletal muscle will be an important breakthrough for future research.

For example, studies in cardiac tissue have revealed that the NEXN protein enhances the stability of SERCA2 by promoting its SUMOylation modification (Guo et al., 2025); this post-translational regulatory pattern has been verified in vascular calcification models, but whether it exerts a similar protective effect in skeletal muscle atrophy or fibrosis remains unknown. Another important finding comes from the field of neurodegenerative diseases: the loss of SERCA2 expression in retinal neurons after ischemic injury directly leads to impaired Ca^2+^ clearance and ER stress, while AAV-mediated SERCA2 gene supplementation can reverse cell death and restore visual function (Shiga et al., 2024). The success of this “gene replacement - functional repair” strategy provides a directly translatable therapeutic path for hereditary skeletal muscle myopathies, but the unique blood supply barrier and fiber type heterogeneity of skeletal muscle may significantly affect intervention outcomes.

However, the translation of these cross-tissue findings to the skeletal muscle field is not straightforward. The core challenge lies in the fact that skeletal muscle, as a metabolic organ with both contractile and endocrine properties (Yi et al., 2025; Smith et al., 2023), its SERCA2 function is dynamically regulated by exercise patterns, nutritional status, and neural innervation, far exceeding the single stimulus response mode of cardiomyocytes or neurons. For example, liver research has identified APOL2 as a novel interacting protein of SERCA2, which affects glucose and lipid metabolism by regulating the ER stress PERK-HES1 axis (Gan et al., 2025), but whether there are similar tissue-specific regulatory factors in skeletal muscle and how they integrate exercise signals with metabolic demands require systematic screening. At the technical level, current skeletal muscle research mostly relies on C2C12 cell lines and gene knockout mice, lacking advanced models that can simulate the three-dimensional structure and metabolic microenvironment of human muscle fibers, which limits the verification of high-precision phenotypic data obtained from cardiomyocyte/neuron research in skeletal muscle.

Beyond mechanism exploration, the clinical translation bottleneck of existing therapeutic strategies is also prominent. Although SERCA activators such as CDN1163 have shown broad-spectrum efficacy in animal models, their lack of tissue selectivity may lead to cardiac toxicity risks. The latest discoveries of subtype-specific regulation provide a solution: the C4orf3 protein in adipose tissue acts as a “molecular resistor” for SERCA2b, finely regulating UCP1-independent thermogenesis (Auger et al., 2025),while the tumor microenvironment reprograms CD8^+^ T cell metabolism through the MFN2-SERCA2 interaction (Yang et al., 2023). These findings suggest that the development of skeletal muscle-specific SERCA2a or SERCA2b modulators, such as LncEDCH1 mimetic peptides or allosteric ligands targeting the TM11 domain, may be the key to achieving precise intervention. In addition, existing clinical trials overly focus on enhancing SERCA2 function, while ignoring its “metabolic braking” role—excessive activation may lead to energy waste through uncoupled thermogenesis, which has been initially revealed in the phenomenon of downregulated SERCA2 expression induced by high-intensity training in exercise adaptation research.

In conclusion, future research on SERCA2 in skeletal muscle needs to move towards “bidirectional translation”: on one hand, the axis mechanism of “ER stress - Ca^2+^ homeostasis - cell survival” refined from cardiac and neurological diseases should be re-verified in skeletal muscle-specific pathological models, such as exercise-induced rhabdomyolysis and age-related sarcopenia; on the other hand, the unique metabolic regulation discoveries in skeletal muscle should also feed back to other fields, providing systemic intervention targets for metabolic diseases. By breaking tissue barriers and establishing a cross-organ SERCA2 functional map can we achieve a clearer link from molecular mechanism to precise treatment.
